# Fitness costs in the presence and absence of insecticide use explains abundance of two common *Aedes aegypti kdr* resistance alleles found in the Americas

**DOI:** 10.1371/journal.pntd.0011741

**Published:** 2023-11-01

**Authors:** Juan J. Silva, Cera R. Fisher, Anastacia E. Dressel, Jeffrey G. Scott

**Affiliations:** Department of Entomology, Comstock Hall, Cornell University, Ithaca, New York, United States of America; Kenya Agricultural and Livestock Research Organization, KENYA

## Abstract

*Aedes aegypti* is the vector of viruses such as chikungunya, dengue, yellow fever and Zika that have a critical impact on human health. Control of adult mosquitoes is widely done using pyrethroids, but resistance has reduced the effectiveness of this class of insecticides. Resistance to pyrethroids in mosquitoes is commonly due to mutations in the *voltage-gated sodium channel* (*Vgsc)* gene (these mutations are known as *knockdown resistance*, *kdr*). In the Americas and the Caribbean, the most common *kdr* alleles are *410L+1016I+1534C* and *1534C*. In this study, we conducted a population cage experiment to evaluate changes in the allele and genotype frequencies of the *410L+1016I+1534C* allele by crossing two congenic strains; one carrying the *410L+1016I+1534C* and another with the 1534C allele. Changes in allele frequencies were measured over 10 generations in the absence of insecticide exposure. We also applied one cycle of selection with deltamethrin at F_9_ to evaluate the changes in allele and genotype frequencies. Our findings indicate that fitness costs were higher with the *410L+1016I+1534C* allele, relative to the *1534C* allele, in the absence of deltamethrin exposure, but that the *410L+1016I+1534C* allele provides a stronger advantage when exposed to deltamethrin relative to the *1534C* allele. Changes in genotype frequencies were not in Hardy-Weinberg equilibrium and could not be explained by drift. Our results suggest the diametrically opposed fitness costs in the presence and absence of insecticides is a reason for the variations in frequencies between the *410L+1016I+1534C* and *1534C* alleles in field populations.

## Author summary

Insecticide resistance limits our ability to control vectors of human diseases. Resistance to widely used pyrethroid insecticides can occur by mutations in the vo*ltage gated sodium channel* (*Vgsc*) and alleles with these mutations are collectively known as *knockdown resistance* (*kdr*). The frequency of resistance alleles is driven by selection with pyrethroid insecticides, but *kdr* alleles decrease in frequency in the absence of insecticide. The relative fitness of different *kdr* alleles to each other is largely unknown. We show that the *1534C* allele is favored in the absence of pyrethroid, but that the *410L+1016I+1534C* allele is favored when deltamethrin selection occurs. These results help to explain the relative frequency of these alleles that have been detected in field collections.

## Introduction

*Aedes aegypti* is the vector of viruses that have devastating impacts on public health and insecticide control of adult mosquitoes is most commonly carried out using pyrethroids. *A*. *aegypti* is the vector of viruses such as chikungunya, dengue, yellow fever and Zika [1–5]. To reduce the burden caused by these diseases, control of adult mosquitoes is mainly done using a class of insecticides known as pyrethroids, with deltamethrin being a common pyrethroid applied for control of *A*. *aegypti* in the field. Pyrethroid toxicity occurs due to the binding and disruption of the voltage-gated sodium channel (VGSC).

Resistance to pyrethroids in mosquitoes is commonly due to mutations in the *Vgsc* gene (mutations that cause pyrethroid resistance are known as *knockdown resistance* or *kdr*). There are 10 *kdr* alleles in *A*. *aegypti* based on the sequencing of either full-length cDNA or PCR products of domains I, II or III of the *Vgsc* gene *(S1 Table)* [6–15]. The most common *kdr* alleles found in the Americas are *410L+1016I+1534C*, and *1534C* [6,8,15,16] (amino acid numbering based on *Musca domestica* VGSC, GenBank: CAA65448.1). The *1534C* allele confers 7- to 16-fold resistance to pyrethroids [17]. The *410L+1016I+1534C* allele confers similar levels of resistance (compared to *1534C*), except that the *410L+1016I+1534C* allele gives higher levels of resistance to deltamethrin and flumethrin [18].

Relative to insecticide susceptible alleles, resistance alleles commonly have a fitness cost in the absence of insecticide exposure [19]. However, field populations often include multiple resistance alleles. Thus, the fitness of the different resistance alleles to each other (in both the presence and absence of insecticide) is important to understanding the evolution of resistance. For example, in *Musca domestica* the relative fitness in the presence of pyrethroids of the different *kdr* alleles is *super-kdr* (M918T+L1014F) > *kdr* (L1014F) > *kdr-his* (L1014H), but in the absence of insecticide the relative fitness is *susceptible* > *kdr-his* > *kdr* > *super-kdr* [20], suggesting that alleles with multiple mutations might have a greater fitness cost than those with a single mutation. In *A*. *aegypti*, the relative fitness costs of different *kdr* alleles (to other *kdr* alleles) is much less studied. This is problematic because many resistance alleles coexist in field populations, and some potentially impose more detrimental disadvantages relative to other resistance alleles in the absence of insecticide exposure. Therefore, it is valuable to understand not only the relative fitness of *kdr* alleles relative to the susceptible alleles, but also to understand the relative fitness of the different *kdr* alleles to each other. Such information is needed to better understand the patterns of alleles found in field populations.

In this study, we investigated the relative fitness of two *A*. *aegypti kdr* alleles (*410L+1016I+1534C* and *1534C*) relative to each other, in the absence and presence of insecticide exposure. We wanted to assess whether a resistance allele with multiple mutations imposed greater fitness costs relative to the allele with only one mutation (in accordance with what was previously found in *M*. *domestica*). Overall, the *410L+1016I+1534C* allele imposes both an astonishing fitness cost in the absence of insecticide, and a strong advantage relative to *1534C* in the presence of deltamethrin exposure.

## Materials and methods

### Mosquito strains

Two congenic strains of *A*. *aegypti* were used in this study. Both strains share the same pyrethroid-susceptible background from the Rockefeller strain (ROCK), but carry different *kdr* alleles. The LMRKDR:ROCK (LKR) is a pyrethroid-resistant strain which is congenic to ROCK, but contains the *kdr* allele *410L+1016I+1534C* [18]. The 1534C:ROCK is a pyrethroid-resistant strain which is congenic to ROCK, but contains the *F1534C* allele [17]. In both congenic strains, the *kdr* alleles are the only mechanism of resistance to pyrethroids [17,18].

### Allele competition experiments

**Fig 1:**
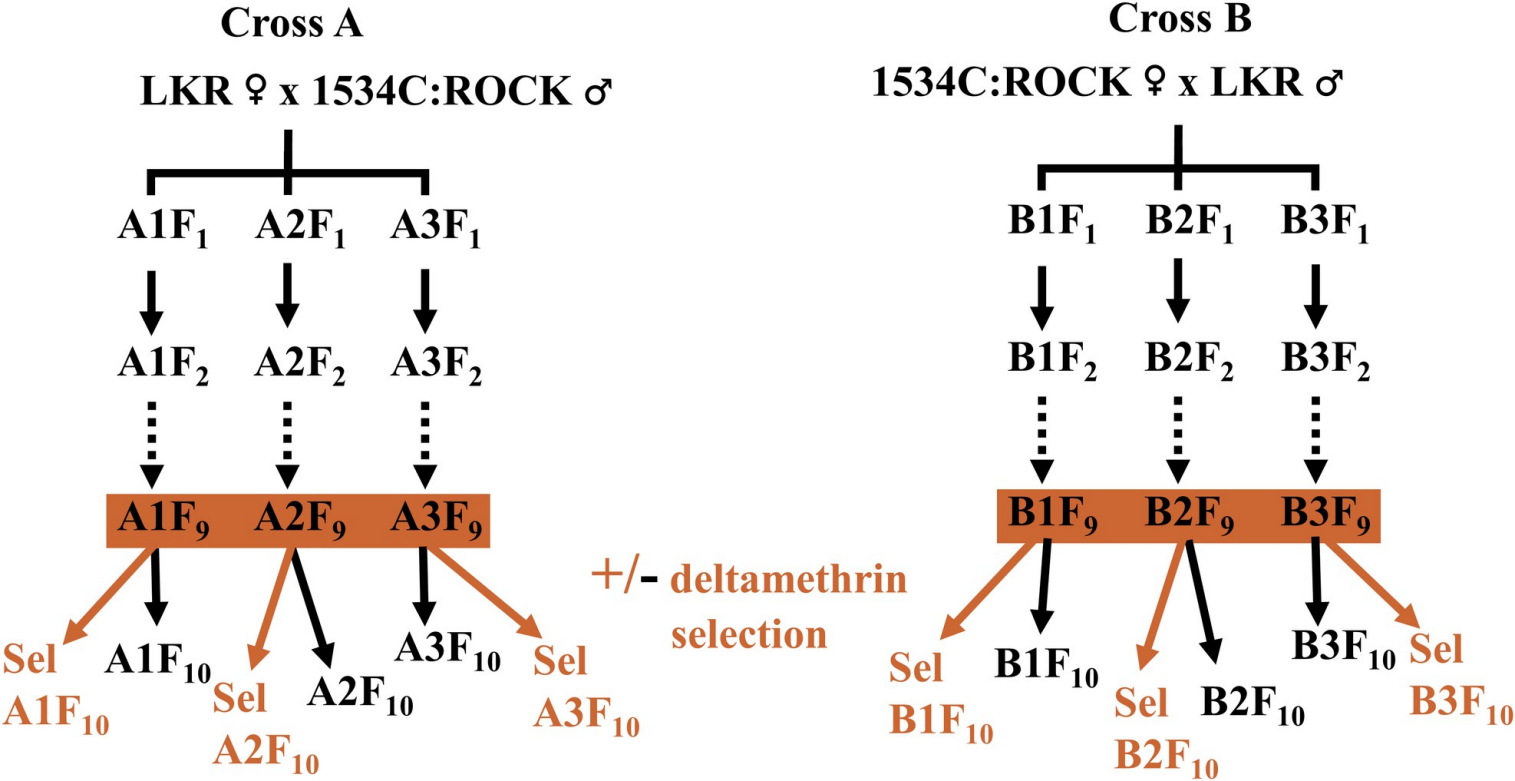
Diagram of the experimental design used.

To conduct the allele competition experiments (also known as population cage experiments), the two congenic strains were crossed: Cross A (LKR females x 1534C:ROCK males) and reciprocal cross B (1534C:ROCK females x LKR males)(Fig 1). For each reciprocal cross, 400 unmated females and 200 males were released into a cage and were allowed to mate *en masse* for seven days. The offspring resulting from crosses A and B were split into three cages (A1, A2, A3, B1, B2 and B3) as shown in Fig 1. Mosquitoes were reared at 25.0–28.5°C (average = 25.6), 26.8–54.5% (average = 47.9%) relative humidity, and a 14:10-h (light/dark) photoperiod. All replicates were run at the same time. Mosquitoes were reared as previously described [21]. Approximately 800 pupae from the larval containers were placed into cages for each following generation. Additional emerging males and females were stored separately in 2 ml Eppendorf tubes at -80°C until they were genotyped.

The genotyping of the mosquitoes was done by allele specific polymerase chain reaction (ASPCR) using primers that were allele specific at codon 1016 of the *Vgsc* (one allele has *410L+1016I+1534C* and allele *1534C* has *V410+V1016+1534C*). The genomic DNA from mosquito heads was extracted as previously described [22] and ASPCR was done using the following thermocycler conditions: For the *1534C* allele, 94°C for 3 min, 36 cycles of 94°C for 30 sec, 62°C for 30 sec and 72°C for 1 min, and 72°C (7 min). For the *410L+1016I+1534C* allele, 94°C for 3 min, 34 x (94°C for 30 sec, 65°C for 30 sec, 72°C for 1 min) and 72°C (7 min). We used 1 μL of extracted DNA as templates for ASPCR. The primers used for each PCR reaction are shown in S2 Table. Each PCR reaction was evaluated on a 1% agarose gel and was scored as homozygous V1016 (PCR band only with V1016 primers), homozygous for 1016I (ASPCR band only with 1016I primers) or heterozygous (PCR band with both 1016V and 1016I primers). For each biological replicate, 45 males and 45 females were genotyped. Every PCR plate included control DNA from 2 samples of LKR, 1534C:ROCK and F_1_ (control DNA was previously sent for sequencing to validate the expected codon V1016I for each corresponding strain or F_1_). In addition to the controls, the codon V1016I was verified for 16 samples from A2F5 and A1F10, eight samples each). The sequencing results for the controls and samples agreed with the results from the gels. Each sample was genotyped using two technical replicates by running PCR reactions and gels twice.

The genotyping results were scored independently by three judges (JJS, JGS and CRF). Each judge assigned a genotype for each mosquito in both technical replicates. If the technical replicates agreed, a final genotype was assigned. A consensus genotype was determined if there was agreement between two out of three judges. If three judges disagreed on the final genotype, then those samples were considered for a third technical replicate. Third replicates were done if there were more than 10 samples per biological replicate for which there was no consensus genotype; otherwise, these samples were dropped from analysis.

### Selection of F_9_ using deltamethrin

We selected 3-7-day old males and females with deltamethrin. The doses used for males and females (0.039 ng/mosquito and 0.078 ng/mosquito, respectively) were chosen to give approximately 80% kill. To conduct the selections, mosquitoes were knocked down with CO_2_ and placed in a paper cup on a bucket filled with ice to keep them anesthetized.

Deltamethrin in acetone (AF10-1Sel, AF10-2Sel, AF10-3Sel, BF10-1Sel, BF10-2Sel and BF10-3Sel populations) or acetone only (AF10-1, AF10-2, AF10-3, BF10-1, BF10-2 and BF10-3 populations) was applied topically to each mosquito with a PB600-1 repeating dispenser and a 10 μL Hamilton syringe (Hamilton Company, Reno NV). Treated mosquitoes (40) were placed in a paper cup covered with nylon tulle and a cotton ball saturated with distilled water and held at 25°C for 24 hours. Both male and unmated female survivors were released in disposable cages (dimensions: A-15.7 x 15.7 x 24 inches, Restcloud Chengdu YiShouWeiSheng Technology Co., Ltd, China) and allowed to mate *en masse*. These mosquitoes were reared as described above and their offspring harvested for *Vgsc* genotyping.

### Allele competition data analysis

Differences between allele and genotype frequencies across generations were tested using linear mixed models and checked for significance from F-values generated from ANOVA in R as previously described [21].

Deviation from Hardy-Weinberg equilibrium (HWE) within generations was assessed using a chi-square test (χ2) to compare the observed genotype counts relative to the expected genotype counts calculated using the allele frequencies of the same generation [21]. The level of significance for statistical analyses was p < 0.05. Simulations were used to evaluate the likelihood of the observed allele frequency changes due to genetic drift. Simulations were done with R software [23] and scripts applied as in a previous study [24]. The model assumed a diploid and panmictic population with a fixed size of 800 individuals and the initial *410L+1016I+1534C* allele frequency for each generation was defined as the observed frequency in the previous interval. Simulations were repeated 50,000 times per generation and p-values were estimated as the number of simulations in which ending allele frequency was equal or more extreme than the initial value of the interval divided by the total number of simulations. The null hypothesis assumed all fluctuations in allele frequencies could be explained by genetic drift. The p-values obtained for the HWE and genetic drift were adjusted for multiple comparisons using Holm’s test [25].

## Results

There were no significant differences in the allele frequencies over generations between reciprocal crosses A and B (Fig 1) for the unselected (ANOVA, p-value = 0. 951) or deltamethrin-selected generations (ANOVA, p-value = 0.174). There were no significant differences in the allele frequencies over generations between males and females in the absence (ANOVA, p-value = 0.888) nor presence of deltamethrin selection (ANOVA, p-value = 0.774. For this reason, allele frequencies from crosses A and B (and from males and females) were pooled for further analysis.

**Fig 2:**
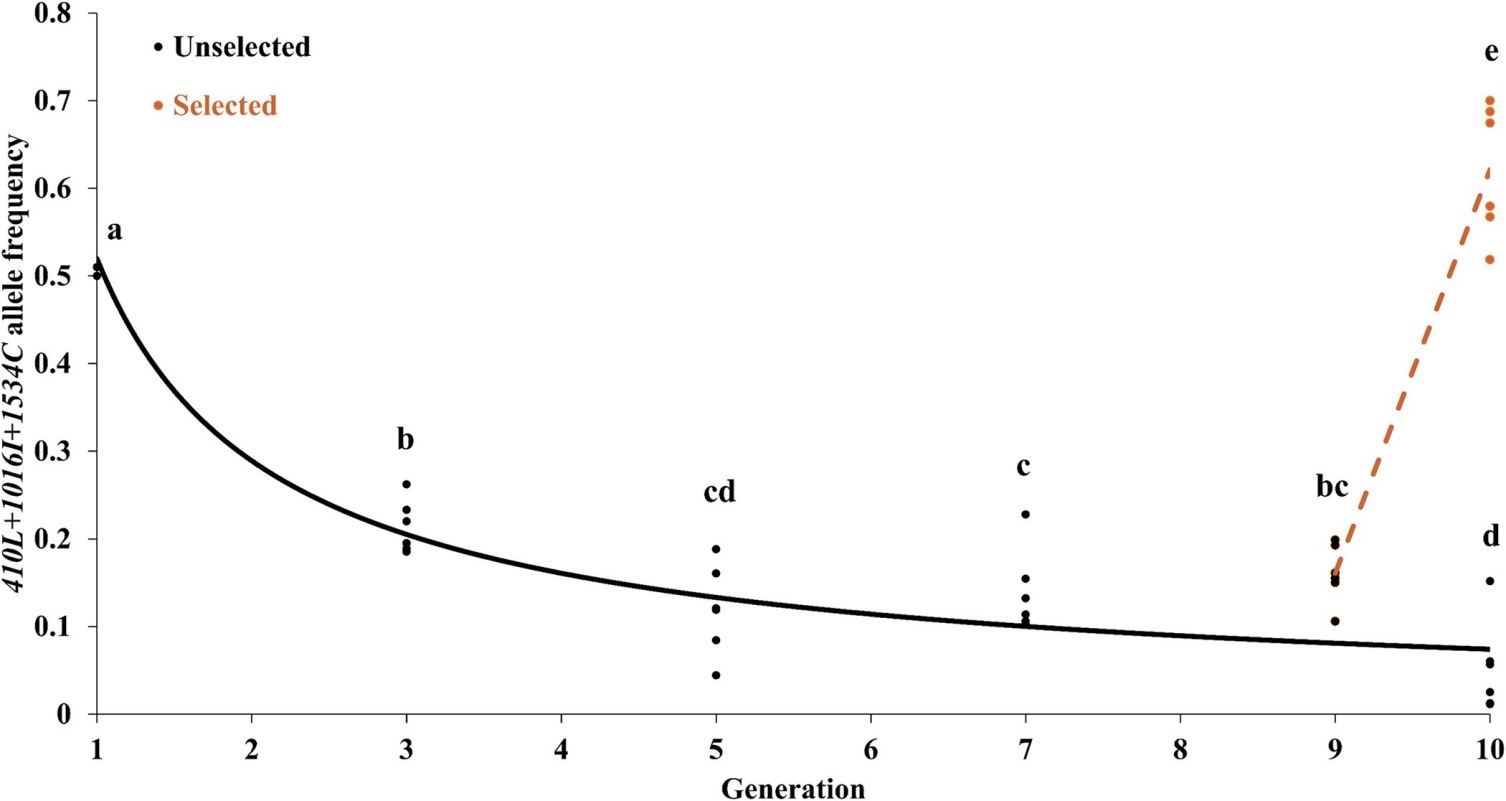
Frequency changes of the *410L+1016I+1534C* allele over 10 generations in the absence and presence of deltamethrin selection. The solid black line represents the changes in allele frequencies in the absence of deltamethrin exposure are represented by a power function model (F_1,34_ = 276.3, r^2^ = 0.89). The dashed orange line represents the changes in allele frequencies from F_9_ to F_10_ in the presence of deltamethrin selection (linear model F_1,10_ = 186.1, r^2^ = 0.95). Different letters represent significant differences based on Tukey’s test.

**Fig 3:**
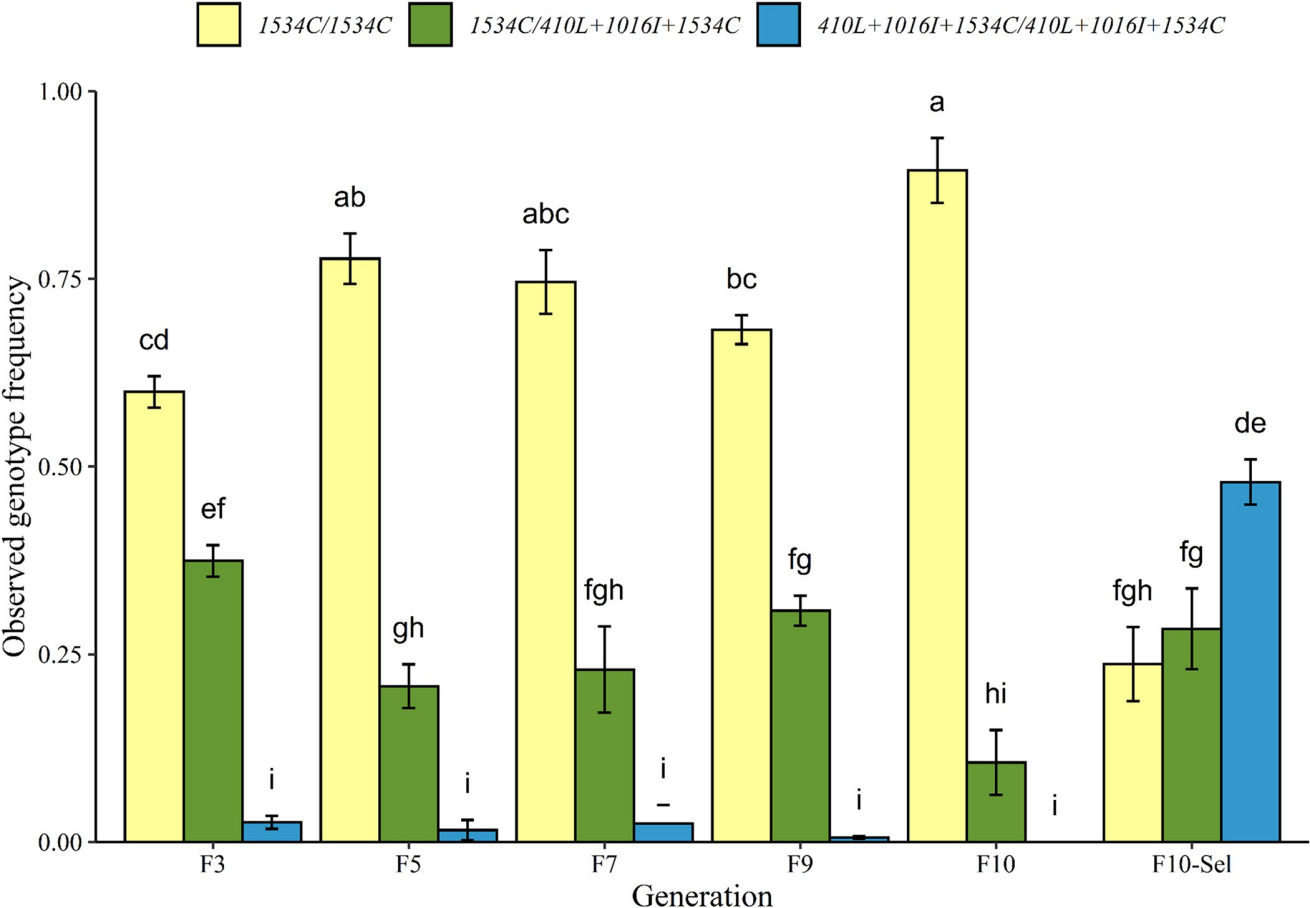
Changes in genotype frequencies over generations in the absence and presence of deltamethrin selection. Different letters represent significant differences based on Tukey’s test.

There was a remarkable fitness disadvantage to the *410L+1016I+1534C* allele, relative to the *1534C* allele, in the absence of insecticide. The fitness cost was most dramatic from the F_1_ to F_3_ and F_5_ where the *410L+1016I+1534C* allele decreased from an average frequency of 50.0% to 21.3% and 11.5%, respectively (S1 Table). The frequency of the *410L+1016I+1534C* allele remained relatively unchanged after the F_5_ (Fig 2 and S1 Table). The frequency of the *410L+1016I+1534C* allele decreased in a trend that is best fitted by a power function model (F_1,34_ = 276.3, r^2^ = 0.89, ANOVA = 2.20x10^-16^). Changes in genotype frequencies over time are shown in Fig 3. The genotype frequencies were not in HWE (within generation) at the F_3_, were largely in HWE in the F_5_ and F_7_, and were variable between replicates for the F_7_ and F_9_ (S1 Table). Our simulations indicated that the changes in allele frequencies from the F_1_ could not be accounted for by drift (all p-values = 0). However, as the changes in allele frequency between generations became more subtle (F_7_-F_10_), drift was found to possibly explain 8 out of 18 results (comparisons made to previous generation) (S1 Table).

The *410L+1016I+1534C* allele exhibited an extraordinarily strong fitness advantage after exposure to deltamethrin with the allele frequency rising from 16% to 62.1% (Fig 2). As expected for a population under selection, the genotype frequencies do not follow the assumptions of HWE (p-values were < 0.0001) (S1 Table). These results are consistent with the observation that the *410L+1016I+1534C* allele gave higher levels of deltamethrin resistance than the 1534C allele [18].

## Discussion

The frequencies of resistance alleles in populations are a function of the level of protection they provide during periods of insecticide exposure, and their relative fitness in the absence of insecticide. Relative to the *1534C* allele, the *410L+1016I+1534C* allele gives similar levels of resistance to common insecticides such as cyhalothrin, cypermethrin and etofenprox (resistance ratios of range from 7.6–14 for these alleles), but gives higher levels of resistance to deltamethrin and flumethrin (resistance ratios of 42 and 57 for the *410L+1016I+1534C* allele, compared to 15 and 13 for the *1534C* allele) [18]. A survey of *A*. *aegypti* populations in the Americas in 2016–2017 found that the *410L+1016I+1534C* allele was more common than the 1534C allele [15]. Data collected from 25 field populations from Brazil (2017–2018) found the *1534C* allele was the most common (40.0%) followed by the *410L+1016I+1534C* allele (38.2%) [16]. In Colombia, (2013–2014) both the *1534C* (43.9%) and *410L+1016I+1534C* alleles (22.2%) were found [6]. In Mexico (2016) the *416L+1016I+1534C* (68.4%) and *1534C* alleles (25.9%) were common [8].

The frequencies of the *410L+1016I+1534C* and *1534C* alleles are strongly influenced by the fitness disadvantage of the *410L+1016I+1534C* allele relative to the *1534C* allele in the absence of insecticides. In contrast in the presence of deltamethrin, the *410L+1016I+1534C* allele has an enormous fitness advantage. During periods of use of pyrethroids such as cyhalothrin (to which the *410L+1016I+1534C* and *1534C* alleles confer similar levels of resistance [17,18]), both alleles would be favored, relative to susceptible alleles. These results bear some similarity to *kdr* alleles in house fly where the *super-kdr* allele (having two mutations) confers higher levels of resistance, but also has a greater fitness cost in the absence of insecticide, compared to *kdr-type* alleles with a single mutation [20]. Given the importance of the VGSC to neurological function, it would be interesting to explore what neurophysiological changes might underline the fitness costs of *kdr* alleles.

The fitness cost of the *410L+1016I+1534C* allele (relative to the *1534C* allele) was remarkably higher than has been found for comparisons of *kdr* versus susceptible alleles. For example, using a similar population cage experiment, the *989P+1016G* [26] and the *F1534C* [21] alleles were shown to have a fitness cost relative to a susceptible allele, but the change in allele frequencies across generations was far less pronounced (frequency of the *kdr* allele decreased in a linear manner and was >0.3 by the F_7_) than we observed in this study. Another study used a strain of *A*. *aegypti* congenic to the pyrethroid-susceptible ROCK strain, but carrying an unknown *kdr* allele (although it was known to carry the V1016I mutation) and measured fitness costs. The frequency of the allele containing the 1016I mutation decreased through 15 generations in the absence of pyrethroid exposure (cross A: r^2^ = 0.527, p = 0.0006 and cross B: r^2^ = 0.569, p = 0.0003, respectively) [27] at a rate similar to what was seen for the *1534C* and *989P+1016G* alleles. It would be helpful to know what the allele was in those experiments, as well as what role (if any) the enhanced detoxification enzymes played in the changes in allele frequency.

Our findings show a strong fitness cost associated with the *410L+1016I+1534C* allele in the absence of insecticide exposure, but this allele provides a fitness advantage when exposed to deltamethrin, relative to the *1534C* allele. Given the dramatic fitness cost of the *410L+1016I+1534C* allele relative to the *1534C* allele in the absence of insecticide, it is possible that the functionality of the VGSC is more drastically impaired for the former allele compared to the latter and investigating the neurophysiological differences between these alleles would be insightful.

## Supporting information

S1 Table Genotype, allele frequencies for each replicate of the LKR x 1534C:ROCK experiment and their associated Hardy-Weinberg equilibrium (HWE) and genetic drift p-values. (DOCX)

S2 Table List of primers used for genotyping the V1016I mutation from LMRKDR:RK and 1534C:ROCK. (DOCX)
