# Supplementary material for: Fitness costs in the presence and absence of insecticide use explains abundance of two common Aedes aegypti kdr resistance alleles found in the Americas
Source: PLoS Negl Trop Dis. 2023 Nov 1;17(11):e0011741. doi: 10.1371/journal.pntd.0011741 (PMC10662748; doi:10.1371/journal.pntd.0011741)
Supplement: S1 Table — (DOCX) [file pntd.0011741.s001.docx]

**Supplementary Table S1.** Genotype, allele frequencies for each replicate of the LKR x 1534C:ROCK experiment and their associated Hardy-Weinberg equilibrium (HWE) and genetic drift p-values.

| Generation | Sample  size | Observed genotype frequency  (expected^#^) | | | Allele frequencies | | HWE^¥^  (p-value) | Genetic drift  (p-value) |
| --- | --- | --- | --- | --- | --- | --- | --- | --- |
|  |  | *1534C/*  *1534C* | *1534C/*  *410L+1016I+1534C* | *410L+1016I+1534C/*  *410L+1016I+1534C* | *1534C* | *410L+1016I+1534C* |  |  |
| AF1-1 | 83 | 0.00 | 1.00 | 0.00 | 0.50 | 0.50 | --- | --- |
| AF1-2 | 89 | 0.00 | 1.00 | 0.00 | 0.50 | 0.50 | --- | --- |
| AF1-3 | 90 | 0.00 | 1.00 | 0.00 | 0.50 | 0.50 | --- | --- |
| BF1-1 | 86 | 0.00 | 0.99 | 0.01 | 0.49 | 0.51 | --- | --- |
| BF1-2 | 88 | 0.00 | 0.99 | 0.01 | 0.49 | 0.51 | --- | --- |
| BF1-3 | 81 | 0.00 | 1.00 | 0.00 | 0.50 | 0.50 | --- | --- |
|  |  |  |  |  |  |  |  |  |
| AF3-1 | 88 | 0.54 (0.59) | 0.44 (0.36) | 0.01 (0.05) | 0.77 | 0.23 | 1.70x10^-10^ | 0.00 |
| AF3-2 | 89 | 0.64 (0.66) | 0.35 (0.30) | 0.01 (0.03) | 0.81 | 0.19 | 2.49x10^-16^ | 0.00 |
| AF3-3 | 82 | 0.52 (0.54) | 0.43 (0.39) | 0.05 (0.07) | 0.74 | 0.26 | 7.68x10^-8^ | 0.00 |
| BF3-1 | 81 | 0.62 (0.61) | 0.33 (0.34) | 0.05 (0.05) | 0.78 | 0.22 | 3.16x10^-13^ | 0.00 |
| BF3-2 | 90 | 0.62 (0.66) | 0.38 (0.31) | 0.00 (0.04) | 0.81 | 0.19 | 2.89x10^-16^ | 0.00 |
| BF3-3 | 82 | 0.65 (0.65) | 0.32 (0.31) | 0.04 (0.04) | 0.81 | 0.19 | 6.66x10^-15^ | 0.00 |
|  |  |  |  |  |  |  |  |  |
| AF5-1 | 81 | 0.69 (0.70) | 0.29 (0.27) | 0.01 (0.03) | 0.84 | 0.16 | 0.68 | 0.00 |
| AF5-2 | 89 | 0.83 (0.84) | 0.17 (0.15) | 0.00 (0.01) | 0.92 | 0.08 | 0.03 | 0.00 |
| AF5-3 | 79 | 0.91 (0.91) | 0.09 (0.08) | 0.00 (0.00) | 0.96 | 0.04 | 9.38x10^-9^ | 0.00 |
| BF5-1 | 87 | 0.76 (0.77) | 0.24 (0.21) | 0.00 (0.01) | 0.88 | 0.12 | 0.12 | 0.00 |
| BF5-2 | 84 | 0.76 (0.78) | 0.24 (0.21) | 0.00 (0.01) | 0.88 | 0.12 | 0.59 | 0.00 |
| BF5-3 | 85 | 0.71 (0.66) | 0.21 (0.31) | 0.08 (0.03) | 0.81 | 0.19 | 0.24 | 1.00 |
|  |  |  |  |  |  |  |  |  |
| AF7-1 | 68 | 0.84 (0.71) | 0.01 (0.26) | 0.15 (0.02) | 0.85 | 0.15 | 1.05x10^-11^ | 1.00 |
| AF7-2 | 90 | 0.54 (0.59) | 0.46 (0.35) | 0.00 (0.05) | 0.77 | 0.23 | 5.89x10^-13^ | 0.00 |
| AF7-3 | 88 | 0.77 (0.79) | 0.23 (0.20) | 0.00 (0.01) | 0.88 | 0.11 | 1.82x^10-4^ | 0.00 |
| BF7-1 | 87 | 0.74 (0.75) | 0.26 (0.23) | 0.00 (0.02) | 0.87 | 0.13 | 1.00 | 0.93 |
| BF7-2 | 85 | 0.79 (0.79) | 0.21 (0.19) | 0.00 (0.01) | 0.89 | 0.11 | 1.00 | 0.91 |
| BF7-3 | 88 | 0.79 (0.81) | 0.20 (0.18) | 0.00 (0.01) | 0.90 | 0.10 | 0.17 | 0.00 |
|  |  |  |  |  |  |  |  |  |
| AF9-1 | 89 | 0.68 (0.72) | 0.31 (0.26) | 0.00 (0.02) | 0.85 | 0.15 | 1.00 | 1.00 |
| AF9-2 | 83 | 0.63 (0.65) | 0.36 (0.31) | 0.01 (0.04) | 0.81 | 0.19 | 1.00 | 0.07 |
| AF9-3 | 88 | 0.60 (0.64) | 0.39 (0.32) | 0.00 (0.04) | 0.80 | 0.20 | 3.6x10^-4^ | 0.00 |
| BF9-1 | 90 | 0.68 (0.70) | 0.32 (0.27) | 0.00 (0.02) | 0.84 | 0.16 | 0.59 | 0.07 |
| BF9-2 | 80 | 0.71 (0.72) | 0.27 (0.26) | 0.01 (0.02) | 0.85 | 0.15 | 1.00 | 0.00 |
| BF9-3 | 85 | 0.80 (0.79) | 0.18 (0.19) | 0.01 (0.01) | 0.89 | 0.11 | 1.00 | 1.00 |
|  |  |  |  |  |  |  |  |  |
| AF10-1 | 80 | 0.95 (0.95) | 0.05 (0.05) | 0.00 (0.00) | 0.97 | 0.03 | 4.7x10^-4^ | 0.00 |
| AF10-2 | 83 | 0.88 (0.88) | 0.12 (0.11) | 0.00 (0.00) | 0.94 | 0.06 | 1.04x10^-3^ | 0.00 |
| AF10-3 | 89 | 0.97 (0.98) | 0.02 (0.02) | 0.00 (0.00) | 0.99 | 0.01 | 7.64X10^-9^ | 0.00 |
| BF10-1 | 88 | 0.88 (0.89) | 0.11 (0.11) | 0.00 (0.00) | 0.94 | 0.06 | 0.01 | 0.00 |
| BF10-2 | 89 | 0.69 (0.72) | 0.30 (0.26) | 0.00 (0.02) | 0.85 | 0.15 | 1.00 | 1.00 |
| BF10-3 | 81 | 0.97 (0.98) | 0.02 (0.02) | 0.00 (0.00) | 0.99 | 0.01 | 6.4x10^-3^ | 0.00 |
|  |  |  |  |  |  |  |  |  |
| AF10-1 Sel^ | 86 | 0.16 (0.12) | 0.32 (0.44) | 0.51 (0.45) | 0.33 | 0.67 | 3.28x10^-201^ | 0.00 |
| AF10-2 Sel | 88 | 0.25 (0.18) | 0.34 (0.49) | 0.41 (0.34) | 0.42 | 0.58 | 3.84x10^-75^ | 0.00 |
| AF10-3 Sel | 80 | 0.24 (0.09) | 0.15 (0.43) | 0.61 (0.47) | 0.31 | 0.69 | 1.44x10^-149^ | 0.00 |
| BF10-1 Sel | 20 | 0.05 (0.09) | 0.50 (0.42) | 0.45 (0.49) | 0.30 | 0.70 | 9.55x10^-33^ | 0.00 |
| BF10-2 Sel | 81 | 0.39 (0.23) | 0.17 (0.49) | 0.43 (0.27) | 0.48 | 0.52 | 1.23x10^-133^ | 0.00 |
| BF10-3 Sel | 89 | 0.33 (0.19) | 0.21 (0.49) | 0.46 (0.32) | 0.43 | 0.57 | 0.00 | 0.00 |

^#^Expected genotype frequencies based on the allele frequencies from the same generation.

^¥^The p values of the HWE are based on the genotypes of the previous generation.

^Sel = selected with deltamethrin.
