# Supplementary material for: Fitness costs in the presence and absence of insecticide use explains abundance of two common Aedes aegypti kdr resistance alleles found in the Americas
Source: PLoS Negl Trop Dis. 2023 Nov 1;17(11):e0011741. doi: 10.1371/journal.pntd.0011741 (PMC10662748; doi:10.1371/journal.pntd.0011741)
Supplement: S2 Table — (DOCX) [file pntd.0011741.s002.docx]

**Supplementary Table S2.** List of primers used for genotyping the V1016I mutation from LMRKDR:RK and 1534C:ROCK.

| **Allele** | **Primer name** | **Sequence (5’ to 3’)** |
| --- | --- | --- |
| 1016V | E20-Sus1 | CATGATCGTGTTCCGGGTATTG |
|  | V1R ARMS | CAAAAGCAAGGCTAAGAAAAGGTTAAGTGC |
| 1016I | E20-Res2 | GCACTCATTCATGATCGTGTTCCGGGTATTA |
|  | I1R ARMS-2 | CAAAAGCAAGGCTAAGAAAAGGTTAAGTATT |
